# Supplementary material for: The microbiome biomarkers of pregnant women’s vaginal area predict preterm prelabor rupture in Western China
Source: Front Cell Infect Microbiol. 2024 Oct 31;14:1471027. doi: 10.3389/fcimb.2024.1471027 (PMC11560878; doi:10.3389/fcimb.2024.1471027)
Supplement: Supplementary file 1 [file DataSheet1.zip › compare_1/Community/Structure_Barplot/phylum/phylum.taxon.Barplot.Legend.With.superkingdom.pdf]

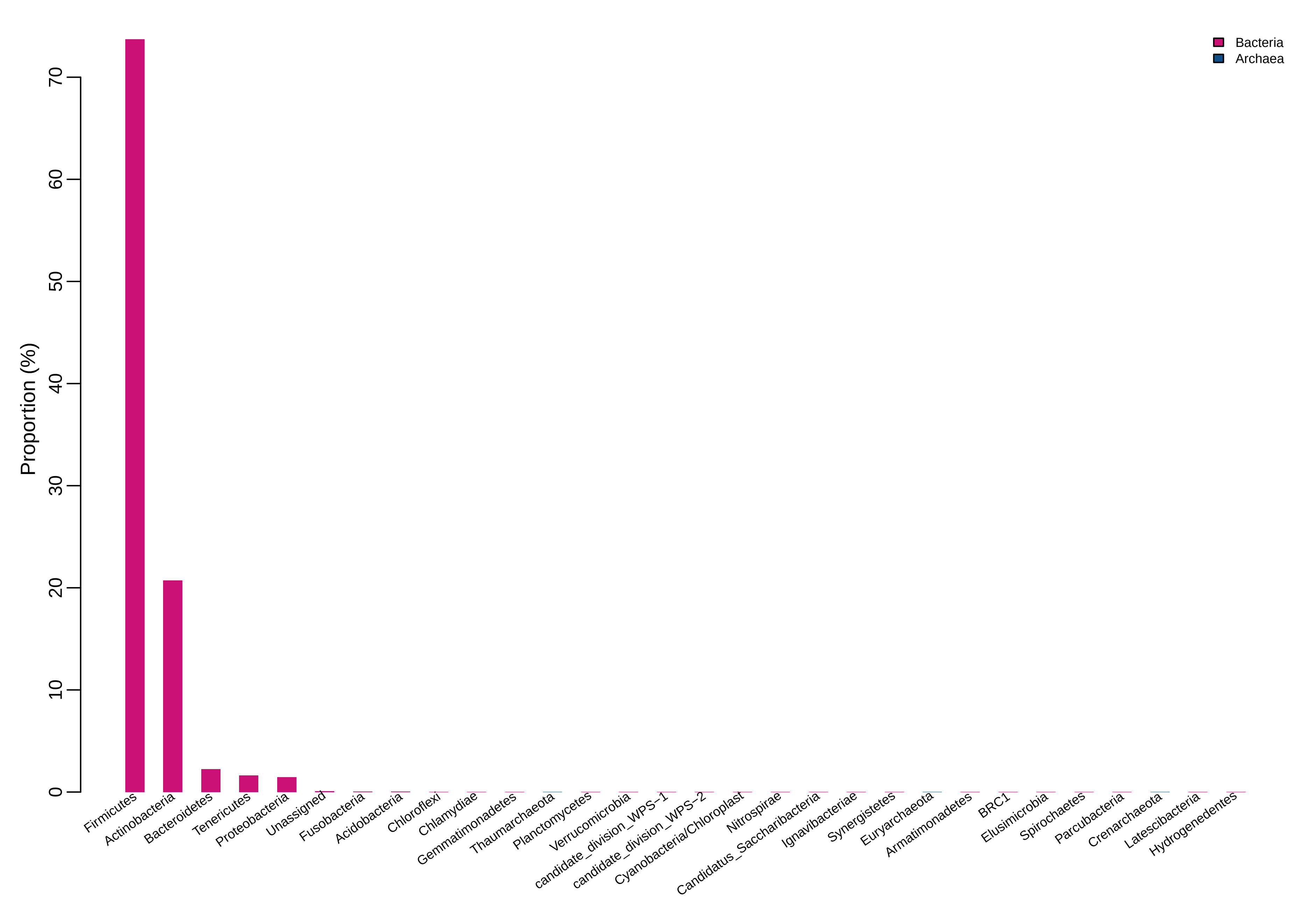

Bacteria
Archaea
Proportion (%)
0
10
20
30
40
50
60
70
Firmicutes
Actinobacteria
Bacteroidetes
Tenericutes
Proteobacteria
Unassigned
Fusobacteria
Acidobacteria
Chloroflexi
Chlamydiae
Gemmatimonadetes
Thaumarchaeota
Planctomycetes
Verrucomicrobia
candidate_division_WPS−1
candidate_division_WPS−2
Cyanobacteria/Chloroplast
Nitrospirae
Candidatus_Saccharibacteria
Ignavibacteriae
Synergistetes
Euryarchaeota
Armatimonadetes
BRC1
Elusimicrobia
Spirochaetes
Parcubacteria
Crenarchaeota
Latescibacteria
Hydrogenedentes
